# Pollination success following loss of a frequent pollinator: the role of compensatory visitation by other effective pollinators

**DOI:** 10.1093/aobpla/plx020

**Published:** 2017-05-27

**Authors:** Allysa C. Hallett, Randall J. Mitchell, Evan R. Chamberlain, Jeffrey D. Karron

**Affiliations:** 1 Department of Biological Sciences, University of Wisconsin-Milwaukee, Milwaukee, WI 53201, USA; 2 Department of Biology, University of Akron, Akron, OH 44325, USA

**Keywords:** *Asclepias verticillata*, *Bombus*, plant-pollinator interactions, pollen receipt, pollination services, pollinator decline, pollinator efficiency, pollinator visitation

## Abstract

Pollinator abundance is declining worldwide and may lower the quantity and quality of pollination services to flowering plant populations. Loss of an important pollinator is often assumed to reduce the amount of pollen received by stigmas of a focal species (pollination success), yet this assumption has rarely been tested experimentally. The magnitude of the effect, if any, may depend on the relative efficiency of the remaining pollinators, and on whether the loss of one pollinator leads to changes in visitation patterns by other pollinators. To explore how a change in pollinator composition influences pollination of *Asclepias verticillata*, we excluded bumble bees from plots in large and small populations of this milkweed species. We then quantified pollinator visitation rates, pollen export and pollen receipt for control plots and for plots where bumble bees were experimentally excluded. We found that exclusion of bumble bees did not reduce pollen receipt by *A. verticillata* flowers. Visitation by *Polistes* wasps increased markedly following bumble bee exclusion, both in small populations (186 % increase) and in large populations (400 % increase). Because *Polistes* wasps were as efficient as bumble bees at pollen transfer, increased wasp visitation offset lost bumble bee pollination services. Thus, loss of a frequent pollinator will not necessarily lead to a decline in pollination success. When pollinator loss is followed by a shift in the composition and abundance of remaining pollinators, pollination success will depend on the net change in the quantity and quality of pollination services.

## Teaser

Sudden declines of frequent and effective pollinators are often assumed to reduce pollination success. Yet this assumption has rarely been tested experimentally and may depend upon responses of other pollinators in the community. We temporarily excluded bumble bees in populations of the milkweed Asclepias verticillata, and quantified pollinator visitation rates and pollen receipt in both control and bumble bee exclusion plots. Asclepias pollination success did not decline following bee exclusion due to a nearly three-fold increase in wasp visitation. This highlights the importance of studies that explore the effectiveness and compensatory responses of other pollinator species in the community.

## Introduction

Nearly 90 % of flowering plant species depend on animal pollinators for pollen transport (Ollerton *et al.* 2011), and plant reproductive success may therefore be sensitive to loss of pollination services (Potts *et al.* 2010; Burkle *et al.* 2013; González-Varo *et al.* 2013). Accumulating evidence suggests that pollinator populations are declining worldwide (Potts *et al.* 2010), and that populations of many bumble bee species have declined considerably both in Europe and North America (Goulson *et al.* 2008; Grixti *et al.* 2009; Cameron *et al.* 2011; Kerr *et al.* 2015). Reductions in pollinator diversity or abundance may influence the amount and source of pollen deposited on stigmas (Aizen and Harder 2007). Pollinator species often differ substantially in their contributions to plant reproductive success due to differences in the number of visits per flower and the amount of pollen deposited per visit (Motten *et al.* 1981; Fishbein and Venable 1996; Ivey *et al.* 2003; Sahli and Conner 2007). Therefore, the severity of the effects of pollinator loss for a focal plant species will depend on the effectiveness of the remaining pollinators and on the subsequent net change in pollination services.

Pollinators can compete for floral resources (Fort 2014), so the loss of an abundant pollinator may release the remaining pollinator species from competition. This may increase visitation rates by less frequent visitors, or may lead to recruitment of additional pollinator species to a focal plant population (Makino and Sakai 2005; Nagamitsu *et al.* 2010; Brosi and Briggs 2013; Song and Feldman 2014). The release of pollinators from competition may thus strengthen existing plant-pollinator interactions or allow new plant-pollinator interactions to form (Memmott *et al.* 2007; Kaiser-Bunbury *et al.* 2010).

How the decline or loss of a pollinator influences plant reproductive success depends on both the lost pollinator’s visitation frequency prior to decline and its pollen transfer efficiency (i.e. the proportion of pollen transferred from an insect’s body to a receptive stigma; Inouye *et al.* 1994, Theiss *et al.* 2007). If a declining pollinator was historically a frequent visitor and efficient at pollen transfer (Vázquez *et al.* 2005; Sahli and Conner 2007), plant reproductive success may decrease unless the pollinator decline is offset by increased visitation from other pollinator species (Rafferty and Ives 2012). Increased visitation by other pollinators may lower, sustain, or even increase the amount of pollen deposited on stigmas (pollination success) following pollinator decline (Madjidian *et al.* 2008), depending on the magnitude of pollinator recruitment and the pollen transfer efficiency of the remaining pollinators.

The effects of pollinator loss may also vary as a function of plant population size. Small populations may have fewer pollinator species (Lamont *et al.* 1993; Rathcke and Jules 1993) and lower visitation rates (Mustajärvi *et al.* 2001). In addition, following pollinator loss, pollinators may preferentially recruit to large populations (Mustajärvi *et al.* 2001), which provide greater floral rewards than small populations. Consequently, loss of a common pollinator may have a differential effect on pollination success in small and large populations (Bernhardt *et al.* 2008).

Here, we explore how a change in pollinator species composition interacts with plant population size to influence pollination success. Bumble bees, wasps and the honey bee *Apis mellifera* are all effective pollinators of whorled milkweed, *Asclepias verticillata* (Kephart 1983; Theiss *et al.* 2007). Preliminary observations in our study sites in 2013 and 2014 indicated that the most frequent visitors to both small and large populations of *A. verticillata* were bumble bees, especially *Bombus griseocollis*. However, bumble bee visitation to *A. verticillata* can be highly variable among sites and among years (e.g. Kephart 1983), and the consequences of this variation are not well known. We therefore used experimental removal of bumble bees to provide insight on the effects of loss of a frequent and effective visitor at our study sites. Our manipulation allows us to address whether bumble bee exclusion: (i) influences the visitation rate of competing pollinator species; (ii) influences whorled milkweed pollination success; and (iii) differentially influences visitation rates and pollination success in small and large populations of whorled milkweed.

## Methods

### Study species

**Figure 1.**
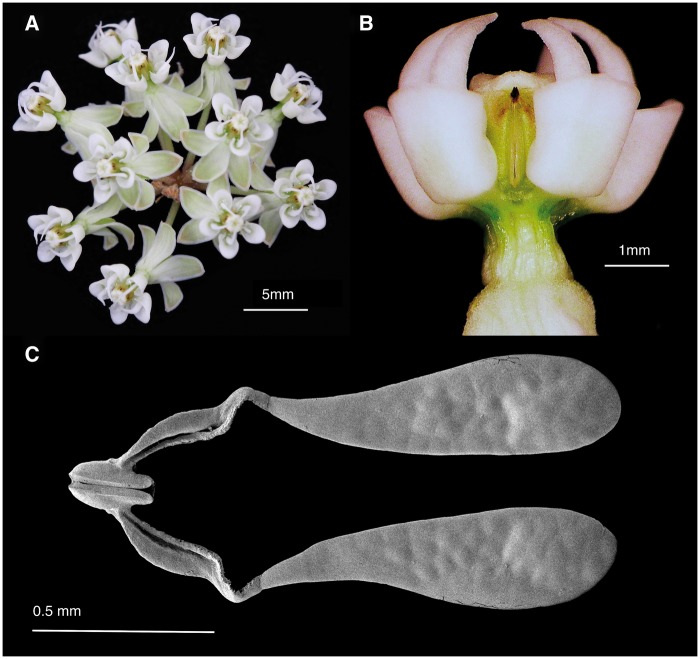
Floral morphology and pollinarium structure for *Asclepias verticillata*. (A) Umbel with 11 flowers. (B) View of a corona showing the black corpusculum of a pollinarium between adjacent tubular hoods. (C) Scanning electron micrograph of an *A. verticillata* pollinarium. The corpusculum is the oval structure between each pollinium.

**Figure 2.**
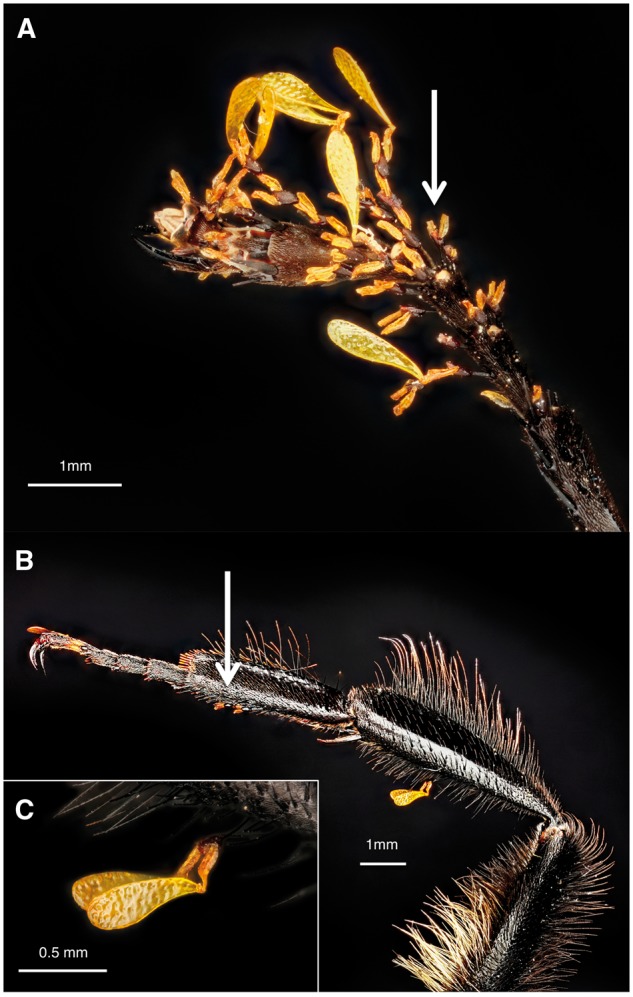
Pollinia and corpuscula loads on (A) a *Polistes* tarsus and (B) a *Bombus* claw, tarsus, and tibia. Pollinia may travel alone or in pairs and are always attached to a corpusculum when on insect legs. The white arrows (A, B) point to corpuscula with remaining translator arms.

*Asclepias verticillata* (Apocynaceae) is a self-incom-patible perennial herb (Kephart 1981) that is pollinated by a diversity of nectar-foraging Hymenoptera (Macior 1965; Willson *et al.* 1979; Kephart 1983; Theiss *et al.* 2007). Plants typically produce a single stem with 2–5 umbels, each with 8–15 small white flowers (Fig. 1A). Each flower has five reflexed petals and five nectar-containing tubular floral hoods (Fig. 1B). Pollen grains are packaged *en masse* in paired saccate pollinia (Kephart and Theiss 2003) (Fig. 1C). Each pollinarium (two pollinia joined via translator arms to a corpusculum) contains 60–75 pollen grains (Wyatt *et al.* 2000, Fig. 1C). Pollinaria are presented in between the floral hoods, for a total of five pollinaria (10 pollinia) per flower. Flowers have two ovaries, each with 30–60 ovules (Wyatt and Broyles 1994; Wyatt *et al.* 2000). Stigmatic chambers are also located in between the floral hoods, for a total of five stigmatic chambers per flower. As a pollinator forages for nectar, its legs settle in between the floral hoods, inadvertently picking up pollinia (Fig. 2). As the pollinator continues foraging, some of the removed pollinia are inserted into stigmatic chambers of flowers on other plants in the population (Macior 1965; Kephart and Theiss 2003; Theiss *et al.* 2007).

### Study populations

We studied six naturally occurring *A. verticillata* populations at the University of Wisconsin-Milwaukee Field Station (Saukville, Wisconsin, USA). All six populations occur within 45 ha of old agricultural fields with sandy soils. These populations are typical habitat for *A. verticillata*, which usually occurs on dry prairies or disturbed areas (Curtis and Green 1949; Kephart 1983). The three ‘small’ populations have 200–300 plants, and the three ‘large’ populations have 3000–4000 plants. *A. verticillata* blooms from early to late August in our study area. We performed our field research during the first three weeks of August 2014, when focal plants were flowering simultaneously.

### Experimental manipulation: bumble bee exclusion

To explore how changes in pollinator composition influence pollination success, we experimentally excluded bumble bees from plots at each study population. We compared pollinaria removal (export) and pollinia insertion (receipt) in both control plots, and in plots with bumble bees excluded. We also monitored pollinator visitation to determine whether visitation rates by other pollinators changed in response to bumble bee exclusion.

In all six populations, we established four plots, each with an area of 0.91 m^2^. No species other than *A. verticillata* were flowering in these plots. In each plot we controlled plant density by removing individual plants to achieve a density of 12 plants m^−2^. In two replicate plots we experimentally excluded bumble bees by gently tapping approaching bees with a 1.2 m white fiberglass rod. This technique chased the bee out of the plot, but did not harm the bee, disrupt other visiting pollinators, or prevent visitation to plants outside the plot. Excluded bees usually left the population, rather than landing on an adjacent plot. The remaining two replicate plots in each population were ‘controls’, had a nearby observer, and did not receive the bumble bee exclusion treatment. In each exclusion plot, an observer prevented bumble bee visitation during the peak period of pollinator visitation: 9:30 to 15:30 local daylight savings time. Bumble bee exclusion and control treatments were performed in three populations at a time over a 4- or 5-day study period. The entire exclusion study lasted 9 days: 4 days for the first set of three populations and 5 days for the second set. For each study period, we chose three populations based on similarity in flowering phenology. We used two large populations and one small population during the first study period, and one large and two small populations during the second study period. All plots were caged outside the window of pollinator exclusion to ensure that no visits occurred. These 0.91 m^3^ cages were fabricated from 2 cm diameter PVC tubing and 0.4 cm fine mesh garden netting. Within each population, all four plots were located within a maximum area of 6 m^2^ and replicate plots were separated by 1–2 m. Plot locations were selected based on similarity of stem density, and to facilitate monitoring of adjacent plots. Prior to caging, we removed flowers that were already open to ensure that flowers collected at the end of the experiment were visited only during the exclusion window.

During bumble bee exclusion and control treatments, we monitored the number of pollinator visits to successive plants in each plot during two 20-minute observation periods each day, for a total of 200 minutes of observation per plot over a 4- or 5-day period. Each ‘visit’ began when a pollinator probed the first flower on a plant, and ended when it flew to another plant. Plots were exposed to pollinators for 6 h on each of 4 or 5 days. Pollinator observation across populations totalled 80 h.

All pollinators were visually identified to genus, and were verified by comparing to specimens in the UW-Milwaukee Field Station collection. Our voucher specimens were then added to the collection. Bumble bees were identified to species, again by referencing the UW-Milwaukee Field Station collection.

### Pollinaria removal and pollinia receipt

At the end of the last day of bumble bee exclusion we sampled *A. verticillata* flowers to quantify pollinaria removal and pollinia receipt. Most flowers were open for 2–5 days during the pollinator exclusion window, and we preferentially collected older flowers to ensure adequate exposure to pollinator visitation. In each of the four plots in all six populations we collected eight flowers from each of two umbels on each of 10 plants. Flowers were frozen until dissection. Using a dissecting microscope, we counted the number of pollinia removed and the number of pollinia inserted for each flower. Pollinators remove pollinia in pairs (each pollinia pair is called a pollinarium), but frequently insert only a single pollinium into a stigmatic chamber (Kephart 1983). The second pollinium often breaks off or remains outside the stigmatic chamber; multiple insertions rarely occur within a single stigmatic chamber. Insertions describe pollination success since, once inserted, pollen grains germinate and grow down the style toward the ovaries.

### Pollinia transport

To better understand the role of bumble bees, wasps and honey bees in pollinia transfer, we collected pollinator specimens near our study populations within one week of completion of the exclusion experiment. Specimens were haphazardly collected from three of the six populations at midday. We sampled at least 19 individuals in each pollinator group, and counted the number of corpuscula and pollinia attached to the mouthparts and legs of each individual using a dissecting microscope. Since no other *Asclepias* species flower concurrently in the area, all corpuscula and pollinia present on collected pollinators were from *A. verticillata*. Because corpuscula remain attached to insects after pollinia deposition, we used corpuscula load to estimate pollen transfer in addition to pollinia load (Kephart and Theiss 2003; Theiss *et al.* 2007). Corpuscula connected to pollinia (Fig. 2) provide a measure of the number of pollinia removed by an individual pollinator. In contrast, corpuscula lacking connected pollinia (Fig. 2A and B) approximate the number of pollinia that have been inserted into *Asclepias* flowers. The ratio of these two variables represents the approximate proportion of pollen transfer (Inouye *et al.* 1994; Theiss *et al.* 2007). Therefore, we could estimate pollinia transfer efficiency as:

[(2×corpuscula load)–pollinia load/(2 × corpuscula load)]

Corpuscula load includes corpuscula without attached pollinia, corpuscula with one pollinium, and corpuscula with two pollinia. Multiplying corpuscula load by two approximates the number of pollinia removed by an insect. Since pollinia load is a measure of individual pollinia, subtracting pollinia load from the number of pollinaria removed accounts for whether one or two pollinia remain attached to a given corpusculum. This measure assumes that pollinia are only inserted into flowers and are not lost during transport or removed by grooming (Theiss *et al.* 2007).

### Data analyses

***Pollinator visitation rates***. Statistical analyses were conducted with SAS v 9.4 and JMP v. 12.0.1 (SAS Institute Inc., Cary, NC, USA). Because visitation rates of different pollinator groups are often correlated we used a MANOVA (Scheiner 2001) to test for overall effects of bumble bee exclusion and population size class on pollinator visitation to *A. verticillata*. We then evaluated the individual constituent ANOVAs to interpret the patterns in the MANOVA. We used type III sum of squares throughout. We first tested whether visitation by the three main pollinator groups (*Bombus*, wasps and *Apis*) was affected by bumble bee exclusion and population size class. To interpret the overall response of wasps to bumble bee exclusion, we then compared visitation among the different wasp visitors (*Polistes*, *Vespula* and *Sphex*). Lastly, in a separate analysis we tested whether overall visitation (total visitation summed across pollinator groups) was influenced by bumble bee exclusion. For each model, we included bumble bee exclusion (control or exclusion) and population size class (small or large) as fixed main effects. We also included a bumble bee exclusion by population size class interaction in the model, as well as population and plot terms. Population was nested within population size class and plot was nested within bumble bee exclusion, population and population size class. The bumble bee exclusion × population size class interaction term determines whether the effect of bumble bee exclusion varied with population size. There were 24 samples in each analysis, representing the mean number of visits to each of the 24 plots (four plots in each of six populations) for each pollinator group. For all visitation models, plot effects were non-significant (*P* > 0.2) so plot was pooled with error. We verified normality and heteroscedasticity of the data.

***Pollinia removal and pollinia receipt***. Because these two response variables are likely to be correlated, we used MANOVAs similar to those above to test for an effect of bumble bee exclusion on pollinaria removal and pollinia receipt. Bumble bee exclusion and population size class were our fixed main effects. We included a bumble bee exclusion × population size class interaction in the model, as well as population and plot terms. Population was nested within population size class, and plot was nested within bumble bee exclusion, population and population size class. There were 230 samples in each analysis, representing pollinaria removal and pollinia insertion for 16 flowers of each of 230 plants (10 plants from each of 23 plots). Of the 24 original plots, one plot (10 plants) was excluded from the analysis because wasps infiltrated the exclusion cage during the experimental window. Plot effects were non-significant (*P* > 0.5) for both removal and receipt models, so plot was pooled with error.

***Pollinia transport***. We used one-way ANOVAs to test for differences in pollinia load, corpuscula load and transfer efficiency between pollinator groups (*Bombus*, *Polistes* and *Apis*). We then performed a post-hoc Tukey’s HSD test to determine which pollinator groups differed significantly from one another. We sampled a total of 80 individual pollinators: *Bombus* [*n* = 24], *Polistes* [*n* = 37] and *Apis* [*n* = 19]. We verified normality and heteroscedasticity of the data. However, pollinia transfer efficiency violated the normality assumption. To improve normality, we reran the model using a Johnson *S*_u_ transformation and found that the results were qualitatively unchanged. We therefore present the untransformed analysis for ease of interpretation.

## Results

### Pollinator visitation rates

**Figure 3.**
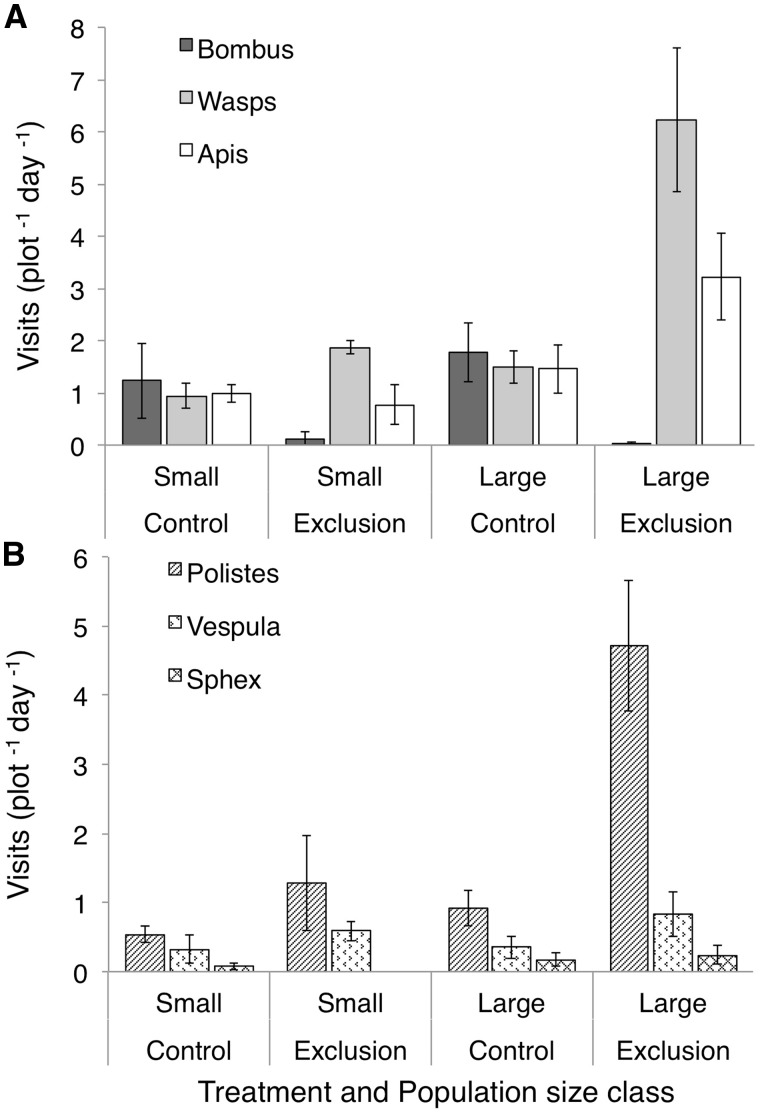
Rate of pollinator visitation to *Asclepias verticillata* flowers in control and bumble bee exclusion plots, and in either small or large populations of *A. verticillata*. (A) Visitation by *Bombus*, wasps, and *Apis*. (B) Visitation by the most common wasp genera composing the wasp category in the first panel, *Polistes*, *Vespula*, and *Sphex*. Bars display means ± SE of plot-level data (*n* = 6 plots/bar, measured over 4 or 5 days depending on population).

In control plots, the three main pollinator groups (*Bombus*, wasps and *Apis*) each represented approximately one third of the total floral visitation to *A. verticillata* (Fig. 3A). 75 % of the *Bombus* visits were by *Bombus griseocollis* and 25 % were by *Bombus vagans*.

In the MANOVA, both *Bombus* exclusion treatment and population size class significantly affected pollinator visitation (Table 1). The individual ANOVAs help to interpret these patterns. *Bombus* exclusion significantly decreased *Bombus* visitation (confirming its efficacy). *Bombus* exclusion also dramatically (and significantly) increased the rate of wasp visitation (293 % overall, regardless of population size, Table 1). The increase in wasp visitation in small populations was 92 %, and in large populations was 313 % (Fig. 3A). Although the MANOVA indicates a significantly higher visitation rate across taxa for larger populations (Table 1 and Fig. 3A), this effect was not significant for any one taxon. The exclusion × population size interaction was significant for the MANOVA, and for the wasp and *Apis* ANOVAs, indicating that the effect of bumble bee exclusion on those taxa varied with population size. For *Apis*, visitation following bumble bee exclusion decreased by 26 % in small populations and increased by 118 % in large populations (Table 1 and Fig. 3A).

**Table 1.** Effect of bumble bee exclusion and population size class on pollinator visitation to *Asclepias verticillata*. We used a MANOVA to test for overall effects. We then used ANOVAs to compare visitation rates by three pollinator groups (*Bombus*, wasps and *Apis*). We calculated mean visitation rate to each of 24 plots (two replicate plots of each treatment per population for six populations). *R*^2^ = 0.59 (*Bombus*), 0.80 (wasps) and 0.68 (*Apis*). Significant values are in bold.

Source	MANOVA	ANOVAs						
			*Bombus*		wasps		*Apis*	
	P for Pilai’s trace (df)	df	*F*	*P*	*F*	*P*	*F*	*P*
*Bombus* exclusion	**0.001** (3,14)	1	**12.24**	**0.003**	**20.31**	**0.0004**	3.30	0.09
Pop size class	**0.0086** (3,14)	1	0.12	0.7	3.45	0.14	3.48	0.14
Exclusion × size	**0.044** (3,14)	1	0.59	0.5	**9.2**	**0.008**	**5.40**	**0.03**
Population (size)	**0.03** (12,48)	4	2.48	0.09	**14.6**	**0.01**	**3.36**	**0.04**
Error		16						

To better evaluate the wasp response we considered the visitation rate for each of the genera making up that group. Again, the MANOVA indicates that all factors significantly affected wasp visitation. The individual ANOVAs indicate that most of the response is determined by *Polistes*, which accounted for 70 % of the increase in wasp visitation (Fig. 3B). Visitation by *Polistes* increased in both small and large populations (Table 2), but the increase was especially pronounced (400 % increase) in large populations (Fig. 3B). Overall visitation showed a strong interaction between bumble bee exclusion and population size class, with an overall increase in visitation upon exclusion (ANOVA; *P* = 0.005) and an even stronger response in larger populations (interaction *P* = 0.0002, Fig. 3A).

**Table 2.** Effect of bumble bee exclusion and population size class on wasp visitation to *Asclepias verticillata*. We used a MANOVA to test for overall effects. We then used ANOVAs to compare visitation by wasps in the genera *Polistes*, *Vespula* and *Sphex*. We calculated mean visitation rate to each of 24 plots (two replicate plots of each treatment per population for six populations). *R*^2^ = 0.81 (*Polistes*), 0.44 (*Vespula*) and 0.71 (*Sphex*). Significant values are in bold.

Source	MANOVA	ANOVAs						
			*Polistes*		*Vespula*		*Sphex*	
	P for Pilai’s trace (df)	df	*F*	*P*	*F*	*P*	*F*	*P*
*Bombus* exclusion	**0.004** (3,14)	1	**23.79**	**0.0002**	3.50	0.08	0.01	0.94
Pop size class	**0.003** (3,14)	1	3.81	0.12	0.20	0.7	1.27	0.3
Exclusion × size	**0.038** (3,14)	1	**10.85**	**0.005**	0.28	0.6	1.55	0.3
Population (size)	**0.006** (12,48)	4	**4.45**	**0.013**	2.09	0.13	**7.12**	**0.002**
Error		16						

### Pollinaria removal and pollinia receipt

**Figure 4.**
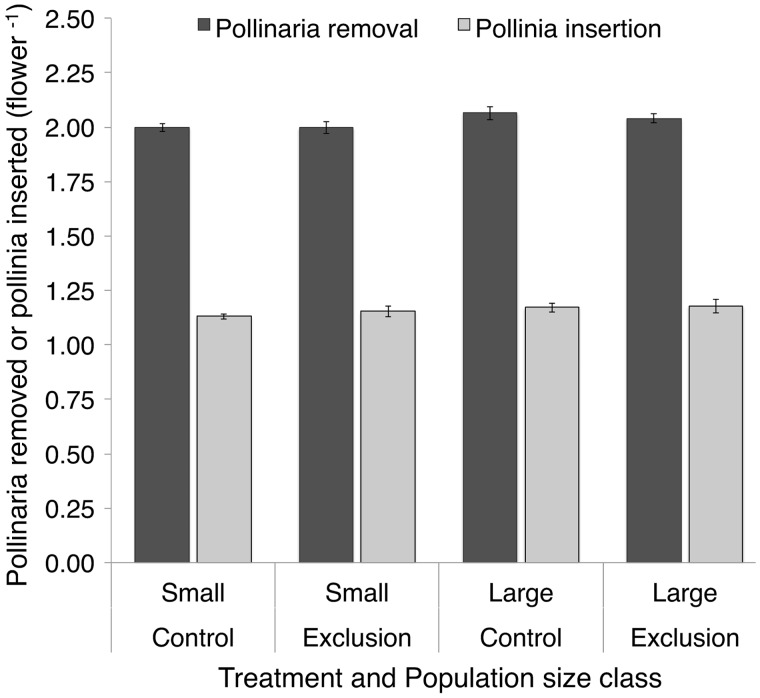
Number of *Asclepias verticillata* pollinaria removed (dark grey) and pollinia inserted (light grey) per flower in control and exclusion plots, and in either small or large populations. Each pollinaria represents two pollinia. Bars display means ± SE of plant-level data (*n* = 60 plants/bar).

Pollination success (pollinaria removal and pollinia receipt per flower) did not vary in response to bumble bee exclusion treatment or population size class (Table 3 and Fig. 4). The constituent responses of the MANOVA are of interest in themselves, so we now consider them. The significant difference in pollinaria removals among population size classes (*P* = 0.02) is quite small and is unlikely to be biologically meaningful (a 2.5 % difference in pollinaria removal between size classes; means and standard errors are 2.04 ± 0.01 and 1.99 ± 0.02 for large and small populations, respectively; Fig. 4). No other effect in the model for either response variable approached significance (Table 3).

**Table 3.** Effect of bumble bee exclusion and population size class on removal (export) and insertion (receipt) of *Asclepias verticillata* pollinia. We used a MANOVA to test for overall effect. We then used ANOVAs to quantify mean pollinaria removal and pollinia receipt for 10 plants (16 flowers per plant) from each of 23 plots (two replicate plots of each treatment per population for six populations, one plot excluded). *R*^2^ = 0.03 for pollinia removal; *R*^2^ = 0.03 for pollinia receipt. Significant values are in bold.

Source	MANOVA	ANOVAs				
			Removal		Insertion	
	P for Pilai’s trace (df)	df	*F*	*P*	*F*	*P*
*Bombus* exclusion	0.34 (2,221)	1	0.33	0.56	0.28	0.60
Pop size class	0.13 (2,221)	**1**	**13.58**	**0.02**	1.70	0.27
Exclusion × size	0.86 (2,221)	1	0.3	0.58	0.19	0.66
Population (size)	0.70 (8,444)	4	0.31	0.87	1.06	0.38
Error		222				

### Pollinia transport

**Figure 5.**
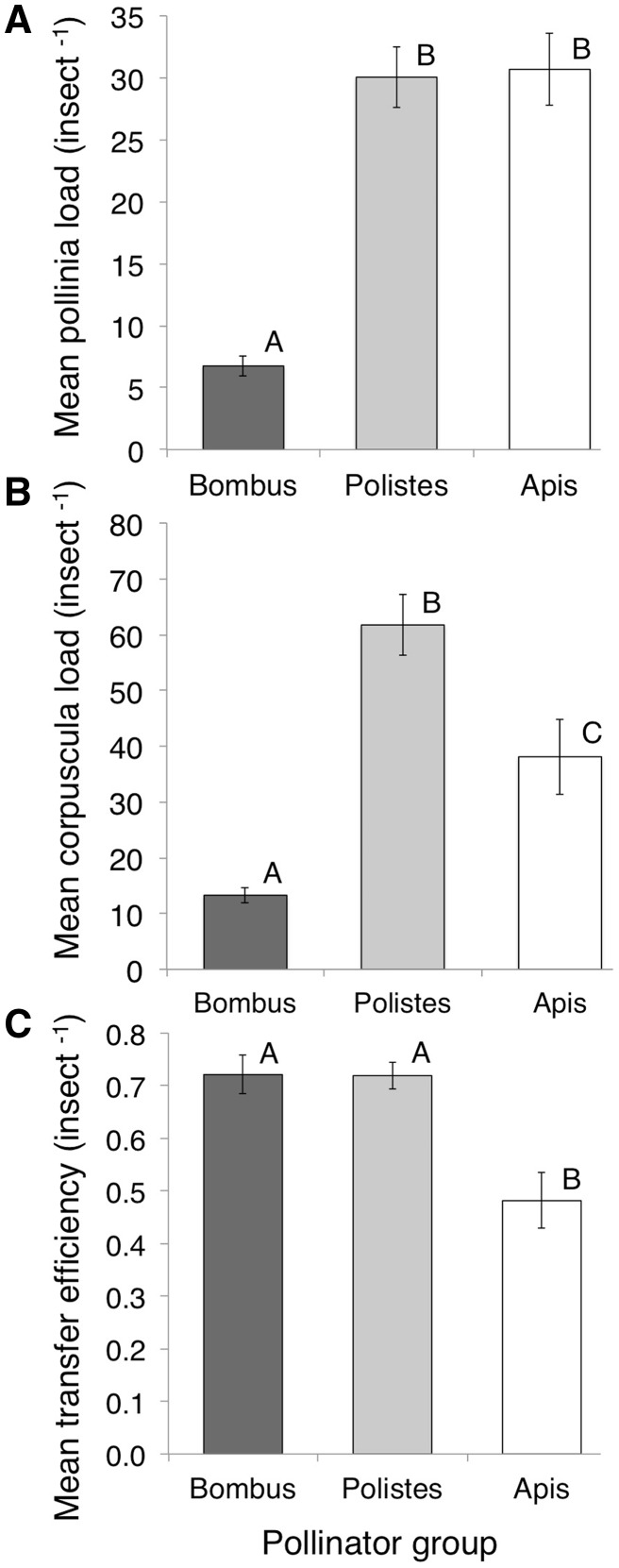
(A) Numbers *of Asclepias verticillata* pollinia per insect across pollinator groups. (B) Numbers of *A. verticillata* corpuscula per insect across pollinator groups. (C) Efficiency of transfer of *A. verticillata* pollinia across pollinator groups. Pollinia transfer efficiency is a measure of the proportion of *A. verticillata* pollinia removed by a pollinator that are missing from corpuscular loads on captured insects, implying potential insertion into other *A. verticillata* flowers. Bars display means ± SE for each pollinator group (*Bombus* [*n* = 24], *Polistes* [*n* = 37], and *Apis* [*n* = 19]). Letters denote significant differences between pollinator groups (post-hoc Tukey’s HSD test).

Pollinia load, corpuscula load and pollinia transfer efficiency differed significantly among pollinator groups (Table 4 and Fig. 5). *Polistes* and *Apis* individuals carried more *A. verticillata* pollinia and corpuscula than *Bombus* (Fig. 5 A,B). However, although *Bombus* carried fewer pollinia and corpuscula, *Bombus* pollinia transfer efficiency was equal to that of *Polistes* (Fig. 5C). *Apis* transfer efficiency was significantly lower than that of both *Bombus* and *Polistes* (Fig. 5C).

**Table 4.** One-way ANOVAs testing for differences amongst pollinator groups in numbers of *Asclepias verticillata* pollinia, numbers of *A. verticillata* corpuscula, and pollinia transfer efficiency of individual insects. Pollinia transfer efficiency is a measure of the proportion of *A. verticillata* pollinia removed by a pollinator that are subsequently inserted into other *A. verticillata* flowers. We sampled a total of 80 individual pollinators (*Bombus* [*n* = 24], *Polistes* [*n* = 37] and *Apis* [*n* = 19]). *R*^2^ = 0.45 (pollinia load), 0.45 (corpuscula load) and 0.24 (pollinia transfer efficiency). Significant values are in bold.

Response	Source	df	MS	*F*	*P*
Pollinia load	Pollinator group	2	4648.45	**32.03**	**<0.001**
	Error	77	145.15		
Corpuscula load	Pollinator group	2	17228.7	**31.55**	**<0.001**
	Error	77	546.1		
Pollinia transfer efficiency	Pollinator group	2	0.4123	**12.48**	**<0.001**
	Error	77	0.0330		

## Discussion

Exclusion of a frequent and efficient pollinator did not reduce *A. verticillata* pollination success. However, wasp visitation increased nearly three-fold following bumble bee exclusion, suggesting that wasps were released from competition, and that increased wasp visitation compensated for lost visitation by bumble bees.

Several recent papers have suggested that pollinator declines are likely to lower pollination success (Biesmeijer *et al.* 2006; Lundgren *et al.* 2013; Thomann *et al.* 2013). In one of the few studies to explore this hypothesis experimentally, Brosi and Briggs (2013) removed the most locally abundant bumble bee species from populations of *Delphinium barbeyi*. The authors found that *Delphinium* reproductive success declined following manipulation, even though plants still received pollination services from several other pollinator species. Our results, in contrast, provide evidence that pollination services offered by competing pollinator species can sometimes offset loss of an abundant pollinator. In our study, increased visitation by wasps preserved pollination success and prevented decline in pollination function despite bumble bee loss. This finding underscores that pollinator losses may not always reduce pollination success, and that a direct link between pollinator decline and reduced plant reproduction should not be assumed. Future research on loss of a focal pollinator should, therefore, explore compensatory responses of other species in the community.

### Variation in space and time

The abundance and species composition of pollinators visiting a focal species may vary in both space and time, and may be influenced by changes in the abundance of other plant species in the community (Alarcón *et al.* 2008; Mitchell *et al.* 2009; CaraDonna *et al.* 2017). In four populations of *A. verticillata* from Indiana (USA), Kephart (1983) reported marked among-population variation in the proportion of visits by bumble bees and wasps. Therefore, the opportunity for other species to compensate for loss of bumble bees might vary spatially, depending on the abundance of wasps near a focal population. The magnitude of the response by wasps might also vary among years. For example, Fishbein and Venable (1996) found strong among-year variation in visitation rate by different taxa pollinating *Asclepias tuberosa*. Rafferty and Ives (2012) also noted substantial temporal variation in the composition of pollinators visiting *Asclepias incarnata*. Therefore, among-year differences in the phenology of plants and pollinators may lead to a substantial decline in pollinator success in some years, but have little to no effect in other years.

Whether a change in the species composition of visitors affects pollination success may depend on the effectiveness of the lost pollinator and the remaining pollinator assemblage. While we found no difference in pollinia transfer efficiency between bumble bees and *Polistes* wasps, increased visitation by *Polistes* wasps compensated for the loss of bumble bee visits. It is unclear why the marked increase in *Polistes* visitation did not result in an increase in *Asclepias* pollination success. One possibility is that while both taxa have similar transfer efficiency, single visits by *Bombus* may be more likely to insert pollinia into a stigmatic chamber than single visits by *Polistes*.

### Effects of population size

The magnitude of pollinator recruitment following decline of a frequent pollinator may also depend on the size of a flowering plant population. We found that more *Polistes* wasps were recruited to large *A. verticillata* populations than to small populations. Since bumble bee exclusion is likely to reduce nectar consumption, increased nectar may be available to other pollinator groups (Thomson 1988). Furthermore, because large plant populations may sustain more pollinator individuals, exclusion plots in large populations may have more foraging *Polistes* individuals nearby than do exclusion plots in small populations. This suggests that pollinator recruitment to small populations may be limited by the number of pollinator individuals, which may restrict the ability of remaining pollinators to offset the effects of pollinator loss.

The present study explored how pollinator loss in local patches influenced visitation by competing pollinators. However, patterns of pollinator visitation and pollination success at this scale may differ from the response to declines across larger landscapes. Following small-scale pollinator loss, the remaining pollinators may readily compensate for the local decline of a common pollinator. In contrast, a landscape wide decline might exhaust the capacity of other pollinators in the region to increase recruitment. Therefore, it is possible that reduction in pollination services following broad, landscape wide declines of bumble bees would not have been offset by recruitment of *Polistes* wasps.

Our findings, in conjunction with those of Brosi and Briggs (2013), suggest that the effects of pollinator decline on pollination success can vary among plant species and ecological contexts. This emphasizes the need for additional experimental studies of the effects of pollinator loss on pollination success. Such studies may be especially important for assessing how changes in pollination services influence populations of rare or endangered plant species, and can also be used to explore the implications of pollinator decline at the community level, especially in fragmented habitats.

## Conclusions

We excluded bumble bees from experimental plots in small and large populations of *A. verticillata*. Our results demonstrate that pollinator loss will not necessarily lead to a decline in pollination success. However, the effects of pollinator decline on pollination success may vary among species, and may even vary among populations. Our work suggests that pollinator declines may shift the composition of visiting pollinator species, and that the consequences of decline or loss may hinge on the net change in quantity and quality of pollination services.

## Sources of Funding

This work was supported by a University of Wisconsin-Milwaukee Research Growth Initiative award to J.D.K. and a grant from Prairie Biotic Research to A.C.H.

## Contributions by the Authors

The experiment was designed by A.C.H., J.D.K., and R.J.M. Field work and microscopy were performed by A.C.H. and E.R.C. All authors contributed to data analyses and writing.

## Conflict of Interest Statement

None declared.
